# Linkage and association analysis of circulating vitamin D and parathyroid hormone identifies novel loci in Alaska Native Yup’ik people

**DOI:** 10.1186/s12263-016-0538-y

**Published:** 2016-08-02

**Authors:** Stella Aslibekyan, Laura K. Vaughan, Howard W. Wiener, Bertha A. Hidalgo, Dominick J. Lemas, Diane M. O’Brien, Scarlett E. Hopkins, Kimber L. Stanhope, Peter J. Havel, Kenneth E. Thummel, Bert B. Boyer, Hemant K. Tiwari

**Affiliations:** 1 Department of Epidemiology, University of Alabama at Birmingham, Birmingham, AL USA; 2 Department of Biostatistics, University of Alabama at Birmingham, Birmingham, AL USA; 3 Department of Health Outcomes and Policy, College of Medicine, University of Florida, Gainesville, FL USA; 4 Department of Biology, King University, Bristol, TN USA; 5 Center for Alaska Native Health Research, Institute of Arctic Biology, University of Alaska Fairbanks, Fairbanks, AK USA; 6 Departments of Molecular Biosciences and Nutrition, University of California at Davis, Davis, CA USA; 7 Department of Pharmaceutics, University of Washington, Seattle, WA USA

**Keywords:** Alaska Native, Vitamin D, Linkage, n-3 fatty acids, Parathyroid hormone

## Abstract

**Background:**

Vitamin D deficiency is a well-documented public health issue with both genetic and environmental determinants. Populations living at far northern latitudes are vulnerable to vitamin D deficiency and its health sequelae, although consumption of traditional native dietary pattern rich in fish and marine mammals may buffer the effects of reduced sunlight exposure. To date, few studies have investigated the genetics of vitamin D metabolism in circumpolar populations or considered genediet interactions with fish and n-3 fatty acid intake.

**Methods:**

We searched for genomic regions exhibiting linkage and association with circulating levels of vitamin D and parathyroid hormone (PTH) in 982 Yup’ik individuals from the Center for Alaska Native Health Research Study. We also investigated potential interactions between genetic variants and a biomarker of traditional dietary intake, the δ15N value.

**Results:**

We identified several novel regions linked with circulating vitamin D and PTH as well as replicated a previous linkage finding on 2p16.2 for vitamin D. Bioinformatic analysis revealed multiple candidate genes for both PTH and vitamin D, including *CUBN*, *MGAT3*, and *NFKBIA*. Targeted association analysis identified *NEBL* as a candidate gene for vitamin D and *FNDC3B* for PTH. We observed significant associations between a variant in *MXD1* and vitamin D only when an interaction with the δ15N value was included. Finally, we integrated pathway level information to illustrate the biological validity of the proposed candidate genes.

**Conclusion:**

We provide evidence of linkage between several biologically plausible genomic regions and vitamin D metabolism in a circumpolar population. Additionally, these findings suggest that a traditional dietary pattern may modulate genetic effects on circulating vitamin D.

**Electronic supplementary material:**

The online version of this article (doi:10.1186/s12263-016-0538-y) contains supplementary material, which is available to authorized users.

## Background

Vitamin D, a steroid hormone synthesized in the skin or acquired through diet, and parathyroid hormone (PTH), secreted by parathyroid glands, work synergistically to promote absorption of dietary calcium and phosphates (reviewed by [31]). In addition, vitamin D affects cell cycle, proliferation, differentiation, and apoptosis through vitamin D receptor response elements found on several hundred genes (reviewed by [40]). As a consequence of its physiological importance, a large body of evidence links circulating vitamin D to numerous health outcomes, including skeletal health, cardiovascular disease, type 1 diabetes, type 2 diabetes, metabolic syndrome, autoimmune diseases, multiple sclerosis, and some types of cancer ([22], reviewed by [15, 16]).

The primary form of circulating vitamin D, 25-hydroxy-vitamin D (25(OH)D), is converted to the metabolically active form 1,25-dihydroxy-vitamin D (1,25(OH)_2_D). The conversion is regulated by PTH as part of a feedback system maintaining calcium and phosphate homeostasis (reviewed by [13, 17, 31]). Concentrations of 25(OH)D in the blood, which aggregately reflect endogenous generation through UVB exposure (D_3_), exogenous dietary intake (D_3_ from animal sources, D_2_ from plant sources), and supplementation, are considered the best indicator of vitamin D status (reviewed by [50]). In addition to the environmental inputs of sunlight and diet, high inter-individual variation in circulating 25(OH)D as well as family studies suggest a role for genetic determinants [1, 14]. Large-scale genome-wide association studies (GWAS) of 25(OH)D have confirmed associations with polymorphisms near cholesterol synthesis, hydroxylation, and vitamin D transport genes in individuals of European descent [1, 46]. Despite the documented importance of ancestry to vitamin D status [42, 46], genetic risk factors for other populations are less established.

Of particular salience is the study of vitamin D in Arctic populations, where exposure to sunlight is greatly reduced for at least half of the year (reviewed in [15]). However, this deficit may be corrected by traditional subsistence diets that are rich in vitamin D due to high intake of fish such as salmon or halibut and marine mammals. Furthermore, there is evidence of physiological adaptations leading to more efficient calcium absorption. For example, in Greenland Inuit people, vitamin D is produced at a lower rate than in Europeans, but the rate of PTH-mediated conversion to 1,25(OH)_2_D is higher [37]. Another study documented normal serum calcium despite low 25(OH)D and dietary calcium in Canadian Inuit children, postulating that evolutionary pressures have selected for vitamin D receptors that bind more strongly to the vitamin D molecule [10, 41]. For these reasons, investigating genetic determinants of vitamin D and PTH in Arctic populations, as well as gene-diet interactions with the intake of vitamin D-rich fatty fish, is likely to yield unique insights.

To that end, we performed the first genome-wide linkage analysis of both 25(OH)D and PTH in a population of Yup’ik people participating in the Center for Alaska Native Health Research (CANHR) study. We followed up our findings with targeted association and gene-diet interaction analyses and used complementary bioinformatic tools to identify putative genetic contributors to vitamin D and PTH homeostasis in this community.

## Methods

### The CANHR population and study sample

The CANHR studies genetic, behavioral, and nutritional risk factors for obesity and related cardiometabolic diseases among Yup’ik people in a community-based setting [29]. Recruitment of Yup’ik families was initiated in 2003 and continues in 11 Southwest Alaskan communities, where all residents are invited to participate, resulting in a convenience sample. The present study sample was comprised of 982 non-pregnant Yup’ik individuals (age range, 13^1^–94) and were informative for genetic linkage analysis; of those, 926 passed quality control checks and had either 25(OH)D or PTH measurements or both. All participants signed informed consent documents, and the study protocols were approved by the Institutional Review Boards of the University of Alaska and the National and Alaska Area Indian Health Service Institutional Review Boards, as well as the Yukon Kuskokwim Human Studies Committee.

### Laboratory measurements

Blood samples were collected from participants after an overnight fast and were immediately processed in the field, stored at −15 °C for up to a week, and then stored at −80 °C at the University of Alaska Fairbanks [5]. Total 25(OH)D was ascertained through a quantitative chemiluminescent immunoassay (ARUP Laboratories, Salt Lake City, UT). This assay accurately quantifies the sum of 25(OH)D_3_ and 25(OH)D_2_. The intra- and inter-assay coefficients of variation (CVs) were 5.5 and 12.7 %, respectively. ARUP participates in the College of American Pathologists Laboratory Accreditation Program and has CLIA (Clinical Laboratory Improvement Amendments) certification through the Centers of Medicare and Medicaid Services. PTH levels were assayed at the University of California at Davis using the DSL-8000 ACTIVE® Intact PTH IRMA Kit (Diagnostic Systems Laboratories, Inc., Webster, TX). The procedure employs a two-site immuno-radiometric assay principle [28]. The intra- and inter-assay CVs were 4.2 and 10.3 %, respectively. Traditional dietary intake was assessed using red blood cell (RBC) nitrogen stable isotope ratios (^15^N/^14^N) as previously described [34] and validated [30]. By convention, isotope ratios were expressed in relative abundance using delta values, as follows: δ^15^N = (^15^N/^14^N_sample_ − ^15^N/^14^N_std_)/^15^N/^14^N_std_ × 1000 ‰, in which the standard is atmospheric nitrogen (^15^N/^14^N = 0.0036765). Analytical precision as assessed using reference materials was within 0.2 ‰.

### Genotyping and quality control

Detailed descriptions of genotyping procedures, pedigree analyses, and data cleaning have previously been published [3]. Briefly, genotyping at 6090 loci was performed at the Center for Inherited Disease Research at Johns Hopkins University using the Illumina Linkage-IV panel (Illumina, San Diego, CA, USA), spanning the entire genome with an average genetic distance of 0.58 cM. Due to residual kurtosis following trait transformation, all linkage analyses involving vitamin D levels used the *t*-distribution option in Sequential Oligogenic Linkage Analysis Routines (SOLAR) to control type I error rate [2].

The genotypic data was subject to several quality control measures, specifically the removal of single nucleotide polymorphisms (SNPs) exhibiting inconsistencies with Mendelian segregation or Hardy-Weinberg equilibrium, prior to whole-genome linkage analysis as previously described [3]. Variants that passed quality checks were used to ascertain a cryptic population substructure, deriving “community group” as a dichotomous covariate indicating proximity to the coast.

### Linkage and association analysis

We estimated the heritability and conducted a whole-genome linkage scan using the variance component approach implemented in the SOLAR program as previously described [3]. All models run in SOLAR included age (quartiles), sex (dichotomous), community group (dichotomous, inland vs. coastal), and δ^15^N value quartiles.

We tested all SNPs under the linkage peaks with a LOD score greater than 1.5 for association with the trait using ASSOC from the S.A.G.E. package, which models the full correlation structure of the pedigree [7, 11]. All association models included the same non-genetic covariates that were used for the linkage analysis: sex, age, community, and δ^15^N value quartiles. A sensitivity analysis additionally adjusted for the season of blood sample collection as a categorical variable. For each SNP, the following two models were run: one containing non-genetic covariates and the SNP genotype as an additive effect and one containing non-genetic covariates, SNP genotype as an additive effect, and the interaction between SNP effect and δ^15^N value quartiles. Likelihood ratio tests were used to compare the models. Corrections for multiple testing were ascertained separately for each linkage peak of interest, calculating the effective number of tested SNPs (i.e., independent SNPs not in linkage disequilibrium (LD)) using spectral decomposition of the correlation matrix [23, 32]. Bonferroni corrections for the effective number of SNPs in each linkage peak were used to determine the number of full model results that met the significance threshold (Additional file 1: Table S1). Full models were defined to include both the additive genotype term and the interactions of that term with n-3 polyunsaturated fatty acids (PUFA) intake.

### Bioinformatic analysis

In order to limit the list of potential genes from regions identified by linkage analysis (LOD score >1.5), we used the SIMWALK2 [43] to phase the genotype data. This analysis indicates the positions of recombination events in the haplotypes of the analyzed individuals. We determined the haplotypes for each individual that had no recombination events and included the position of peak evidence for linkage with the trait of interest. The regions thus defined were examined for the full sample, as well as the subsets limited to both the highest and lowest quartile of the trait of interest. Regions in which less than 5 % of the examined sample experienced a recombination event were included in subsequent bioinformatic analysis. This region was very narrow for chromosome 3, so the threshold was relaxed for that region. Within the regions, potential candidate genes were identified using three complementary algorithms: Ingenuity (Qiagen, Redwood City, CA), TOPPGene Suite [6], and ENDEAVOUR [44]. For all three algorithms, training gene lists were based on the keyword “PTH” or “Vitamin D” in the HuGE navigator [49]. Genes were selected if they attained a HuGE score of at least 0.10 and were related to vitamin D and PTH only. Gene names were converted to Ensembl using BioMart Central ID converter as necessary. The settings for all three algorithms were identical to those in a previous analysis by our group [45]. Genes were considered to be putative candidates if the *P* values for both TOPPGene and ENDEAVOUR were ≤0.005 and if they were shown by Ingenuity to interact with the chosen training genes.

## Results

Descriptive characteristics of the Yup’ik study participants are presented in Table 1. All of the *P* values in this table are based on simple two-sample *t* tests, without taking the pedigree correlation into account. RBC δ^15^N values and circulating PTH were both significantly higher among women, while 25(OH)D levels did not vary by gender. The correlation between serum 25(OH)D and PTH levels was estimated at −0.09. Transformations of both traits reduced residual kurtosis from 9.96 to 0.45 for PTH and from 4.00 to 1.10 for 25(OH)D. Heritability was estimated at 0.43 ± 0.07 for log-transformed PTH (*P* value = 1.2 × 10^−10^) and at 0.54 ± 0.07 for Box-Cox transformed 25(OH)D (*P* value = 2.3 × 10^−18^).

**Table 1:** Descriptive statistics of the study sample (*n* = 926)

	Male	Female	*P* value
*N* ^a^	419 (45)	507 (55)	0.004
Coastal community group^a^	184 (50)	186 (50)	0.92
Inland community group^a^	235 (42)	321 (58)	0.0003
Age, years^b^	36.56 (17.56)	38.07 (17.61)	0.17
δ^15^N, ‰^b^	8.80 (1.51)	9.13 (1.54)	0.0001
Parathyroid hormone, pg/mL^c^	52.7 (24.21–114.84)	65.60 (27.83–154.61)	<0.0001
25(OH)D, ng/mL^c^	34.57 (11.47–83.32)	33.96 (12.21–77.69)	0.56

The overall study sample consists of 926 individuals after QC; however, two were missing values for 25(OH)D and two different individuals were missing values for parathyroid hormone

^a^ *N* = sample size (%), *P* value based on binomial distribution

^b^Mean (SD), *P* value based on two-sample *t* test

^c^Mean (95 % CI), *P* value based on two-sample *t* test

**Fig. 1:**
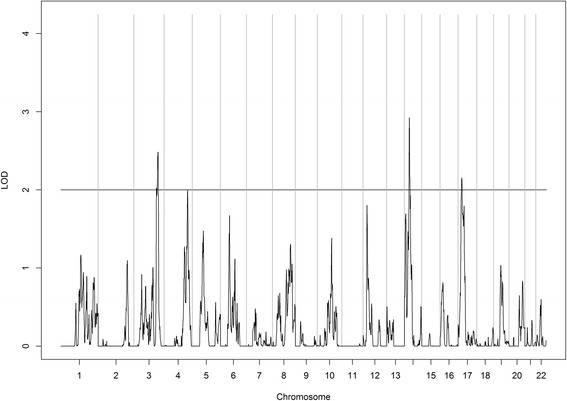
Linkage results for vitamin D. Whole-genome linkage scan for circulating vitamin D in Yup’ik people (*n* = 924). The *X*-axis shows the chromosomal location, and the *Y*-axis displays the LOD score

**Fig. 2:**
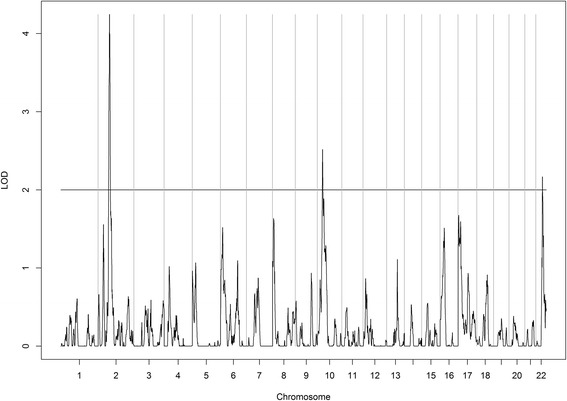
Linkage results for parathyroid hormone. Whole-genome linkage scan for parathyroid hormone in Yup’ik people (*n* = 924). The *X*-axis shows the chromosomal location, and the *Y*-axis displays the LOD score

Results for the genome-wide linkage scan are presented in Table 2 and Figs. 1 and 2. Three regions on chromosomes 2, 10, and 22 yielded a LOD score of at least 2 for 25(OH)D. The linkage peak on chromosome 2 exhibited the strongest signal, with the LOD score exceeding 4 for 25(OH)D. Also, three regions on chromosomes 3, 14, and 17 were linked with PTH with LOD scores greater than 2.

**Table 2:** Linkage peaks with a LOD score >2

Chromosome bands	Peak LOD score	Start SNP	End SNP	Genomic region
25(OH)D				
2p16.2–2p12	4.25	rs1483869	rs741418	52,906,147–75,363,186
10p13–10p12.1	2.52	rs873849	rs638324	14,509,807–27,730,261
22q13.1–22q13.31	2.17	rs138383	rs929090	38,799,861–44,313,849
Parathyroid hormone				
3q25.32–3q26.32	2.48	rs6799097	rs6443567	158,811,068–178,427,716
14q12–14q22.1	2.93	rs7149108	rs735265	32,962,300–52,120,264
17p13.1–17p11.2	2.15	rs9217	rs1037037	7,363,088–18,765,021

After adjusting for multiple testing, two variants on chromosome 2 and one variant on chromosome 10 were significantly associated with 25(OH)D at adjusted *α* = 0.001 and 0.002, respectively, while only one variant on chromosome 3 was significantly associated with PTH at adjusted *α* = 0.001. However, the two variants on chromosome 2 were in perfect LD. Significant SNPs and the associated genes are presented in Table 3. Significant interactions of variants on chromosome 2 with n-3 PUFA intake were observed for 25(OH)D, and there was evidence suggestive of similar interactions between a variant on chromosome 3 and circulating PUFA in the PTH models (Additional files 2 and 3: Figures S1 and S2). Additional adjustment for season did not appreciably change the observed genetic associations (data not shown).

**Table 3:** Significant associations under the linkage peaks

SNP	Chr	Gene	*P* (additive)^a^	*P* (full)^b^	*χ* ^2^ (LRT)^c^	*P* (LRT)^c^
25(OH)D						
rs10205487/rs897119^d^	2	*MXD1*	0.22	0.0006	18.15	0.0004
rs10828183	10	*NEBL*	0.0005	0.004	3.18	0.37
Parathyroid hormone						
rs1353894	3	*FNDC3B*	0.03	0.0008	14.21	0.003

^a^ *P* value for the model including age, sex, community group, δ^15^N quartiles, and additive genotype

^b^ *P* value for the model including all of the above plus the interaction term between the additive genotype and δ^15^N quartiles

^c^Chi-square statistic and *P* value for the likelihood ratio test comparing the additive model with the full model

^d^rs10205487 and rs897119 were found to be in perfect linkage disequilibrium, with identical estimates of association signal

Results of the bioinformatic analysis are summarized in Table 4. None of the a priori identified training genes were located under the linkage peaks. All peaks except for the one on chromosome 2 contained candidate genes proposed by all three bioinformatic algorithms.

**Table 4:** Potential candidate genes identified by bioinformatic analysis of genes within linkage peaks for 25(OH)D and parathyroid hormone

Chromosome bands	Putative candidate genes
25(OH)D	
10p13–10p12.1	*CUBN*, *RSU1*
22q13.1–22q13.31	*APOBEC3F*, *APOBEC3G*, *ATF4*, *CACNA1I*, *CBX7*, *CBY1*, *DDX17*, *DMC1*, *GTPBP1*, *MGAT3*, *PDGFB*, *RPL3*
Parathyroid hormone	
3q25.32–3q26.32	*NLGN1*
14q12–14q22.1	*CFL2*, *FOXA1*, *MYH1*, *NFKBIA*, *NKX2-8*, *PAX9*, *PSMA6*, *SFTA3*, *SNX6*
17p13.1–17p11.2	*GAS7*, *MIPOL1*, *MYH2*, *MYH4*, *RCVRN*

## Discussion

The CANHR Yup’ik study population is ideal for investigation of the genetic determinants of circulating vitamin D and potential gene-diet interactions with n-3 PUFA. Despite low levels of sunlight exposure, mean 25(OH)D levels in our study sample were well within the optimal range currently recommended for the general population [4]. This paradox may be explained by both genetic adaptations as well as the high vitamin D content of the traditional diet of the Yup’ik people, which may buffer the effects of insufficient sunlight at higher latitudes (reviewed by [15, 17]). Indeed, a previous analysis of a CANHR subsample demonstrated that intake of locally harvested foods, namely fatty fish, fish roe, fish liver, and wild game, is correlated with higher levels of 25(OH)D [24]. Yet, another study showed that 25(OH)D mediates PTH levels in our study population [25]. Using genotype data from extended pedigrees, biochemical measurements of 25(OH)D, PTH, and the δ^15^N value (a marker of traditional diet rich in vitamin D), and established biological pathways, this report further illustrates the complexity of vitamin D metabolism in the Yup’ik people.

Three linkage peaks were identified for 25(OH)D and three for PTH. The peaks for the two traits did not overlap, reflecting distinct genetic contributions underlying the weakly correlated 25(OH)D and PTH levels in our study population as well as others [21, 39]. All were novel findings except for the locus on chromosome 2, which was initially reported as linked to 25(OH)D_3_ in Northern European families with asthma [48] and associated with bone mineral density in a large meta-analysis [8]. Few genome-wide linkage studies on 25(OH)D and none on PTH are available to use as a comparison. Interestingly, none of our observed linkage regions contained genes previously reported in genome-wide association studies (GWAS) of 25(OH)D or 1,25(OH)2D, e.g., *GC* on chromosome 4 [1] or *CYP2R1* and *DHCR7* on chromosome 11 [46]. Similarly, the previous GWAS association of loci in the *PTH* gene with circulating PTH was not replicated [27]. The reasons for non-replication may include our genotyping approach, which utilized a linkage-specific panel that was not designed to include all physiologically relevant loci, the uniqueness of our population, and chance. The remaining linkage peaks at 10p13–p12.1 and 22q13.1–13.31 for 25(OH)D and 3q25.32–26.32, 14.12–22.1, and 17p13.1–11.2 for PTH are novel and contain several biologically plausible loci.

Using complementary methods, bioinformatic analysis identified several strong candidate genes located under observed linkage peaks. Of those, the most notable is *CUBN* (cubilin), located on chromosome 10. The product of *CUBN*, cubilin, facilitates megalin-mediated delivery of 25(OH)D_3_ to kidney epithelial cells, impacting circulating levels of the hormone [33]. In both human and animals, mutations causing cubulin dysfunction are associated with abnormal vitamin D metabolism (e.g., urinary excretion of 25(OH)D_3_ in humans) [33]. Another biologically interesting finding from the bioinformatic analysis is *MGAT3* (beta-1,4-mannosyl-glycoprotein 4-beta-N-acetylglucosaminyltransferase), located on chromosome 22. *MGAT3* encodes an enzyme implicated in protein glycosylation and the immune response [20]. Preliminary data illustrate *MGAT3* mRNA as a potential biomarker of response to vitamin D therapy in Alzheimer’s disease, hinting at its translational potential; however, these findings remain to be independently validated [9]. Isoforms of two other potential candidate genes, *CACNA1I* (calcium channel, voltage-dependent, *T* type, alpha 1i subunit) and *PDGFB* (plate-derived growth factor beta polypeptide), have been shown to be regulated by the vitamin D receptor, respectively affecting calcium homeostasis [12] and mitosis [36].

Several strong candidate genes for PTH were also identified based on their involvement in relevant physiological pathways. *NFKBIA* (nuclear factor kappa light polypeptide gene enhancer in B cells inhibitor, alpha) is a member of the NFkB inhibitor family that binds with the product of *REL* (v-rel reticuloendotheliosis viral oncogene homolog, regulated by the vitamin D receptor) to inhibit Rel/NFkB complexes. In a previous transcriptome-wide study, the expression of *NFKBIA* in white blood cells was shown to be decreased by vitamin D supplementation, potentially affecting downstream immune response [18]. Reflecting the pleiotropic functions of the vitamin D/PTH axis, other potential candidate genes were also implicated immune/inflammatory processes (*NKX2*, *PSMA6*, *SNX6*) as well as cancer (*NKX2*, NKX3, *PAX9*).

Extended pedigrees such as those present in the CANHR study provide a powerful framework for targeted association following whole-genome linkage [47]. Targeted association analysis of the observed linkage peaks identified two SNPs in *MXD1* (MAX dimerization protein 1) and one in *NEBL* (nebulette) that were significantly associated with 25(OH)D levels and one in *FNDC3B* (fibronectin type III domain containing 3B) associated with PTH levels. Both *MXD1* and *FNDC3B* are involved in the VDR/RXR activation pathway, the former being regulated by the vitamin D/VDR/RXR complex [38] and the latter suppressing the expression of *RUNX2* [19], which in turn binds to and activates vitamin D/VDR/RXR [35]. On the other hand, *NEBL* has been implicated in calcium homeostasis [26], although a direct link to vitamin D metabolism has not been investigated.

This study presents novel evidence of linkage between several biologically plausible genomic regions and 25(OH)D/PTH in a study population of Yup’ik people with seasonally low exposure to sunlight and high intake of vitamin D-rich foods. These findings are particularly informative in the context of the vitamin D/VDR/RXR pathway, which illustrates the involvement of candidate loci in maintaining the vitamin D/PTH/calcium homeostasis. To our knowledge, this is the first whole-genome linkage study of PTH overall and the first such study of both phenotypes in a circumpolar population. In addition to replicating a previously reported linkage region for 25(OH)D and identifying novel loci for both 25(OH)D and PTH, we present novel evidence of gene-diet interactions with n-3 PUFA intake. Specifically, a variant in *MXD1* was significantly associated with 25(OH)D only after accounting for its interaction with δ^15^N, a marker of n-3 PUFA intake and traditional lifestyle in Yup’ik people. To the best of our knowledge, no other circumpolar cohorts currently have comparable genotypic and phenotypic measurements, limiting our opportunities for external replication. Future studies in high-latitude populations with unique vitamin D dietary sources and requirements are warranted to establish validity of our findings, laying the groundwork for disease-prevention efforts in Arctic communities.

## Conclusions

We provide evidence of linkage between several biologically plausible genomic regions, located on chromosomes 2, 3, 10, 14, 17, and 22, and vitamin D metabolism in a circumpolar population. Additionally, these findings suggest that the traditional dietary pattern of the Yup’ik people, characterized by high intake of n-3 PUFA, may modulate genetic effects on circulating 25(OH)D.

## Abbreviations

1,25(OH)_2_D, 1,25-dihydroxy-vitamin D; 25(OH)D, 25-hydroxy-vitamin D; CANHR, Center for Alaska Native Health Research; CV, coefficient of variation; HWE, Hardy-Weinberg equilibrium; PTH, parathyroid hormone; PUFA, polyunsaturated fatty acids; SNP, single nucleotide polymorphism; SOLAR, Sequential Oligogenic Linkage Analysis Routines

## Additional files

Additional file 1: Table S1. Actual and effective number of SNPs in each linkage region. (DOCX 13.3 kb) Additional file 2: Figure S1. Distribution of predicted mean values (±standard deviation) of transformed 25(OH)D levels for rs10205487 genotypes within n-3 PUFA quartiles (*n* = 924). The *X*-axis shows the n-3 PUFA quartiles, and the *Y*-axis shows Box-Cox transformed 25(OH)D levels, with the colors of the bars corresponding to the genotype. (DOCX 68.9 kb) Additional file 3: Figure S2. Distribution of predicted mean values (±standard deviation) of transformed parathyroid hormone levels for rs1353894 genotypes within n-3 PUFA quartiles (*n* = 924) with the colors of the bars corresponding to genotype. The *X*-axis shows the n-3 PUFA quartiles, and the *Y*-axis shows log-transformed parathyroid hormone levels, with the colors of the bars corresponding to the genotype. (DOCX 17 kb)

## Footnotes

1 There was only one 13 year old individual, who turned 14 within two weeks of enrollment.
