# Supplementary material for: Linkage and association analysis of circulating vitamin D and parathyroid hormone identifies novel loci in Alaska Native Yup’ik people
Source: Genes Nutr. 2016 Aug 2;11:23. doi: 10.1186/s12263-016-0538-y (PMC4971612; doi:10.1186/s12263-016-0538-y)
Supplement: Additional file 1: Table S1. — Actual and effective number of SNPs in each linkage region. (DOCX 13.3 kb) [file 12263_2016_538_MOESM1_ESM.docx]

Supplemental Table 1. Actual and effective number of SNPs in each linkage region.

| **Chromosome** | **Total SNPs** | **Effective SNPs** |
| --- | --- | --- |
| **25(OH)D** |  |  |
| 2 | 55 | 43 |
| 10 | 24 | 21 |
| 22 | 15 | 14 |
| **Parathyroid Hormone** |  |  |
| 3 | 44 | 38 |
| 14 | 38 | 32 |
| 17 | 31 | 29 |
