# Supplementary figures and images for: Linkage and association analysis of circulating vitamin D and parathyroid hormone identifies novel loci in Alaska Native Yup’ik people
Source: Genes Nutr. 2016 Aug 2;11:23. doi: 10.1186/s12263-016-0538-y (PMC4971612; doi:10.1186/s12263-016-0538-y)

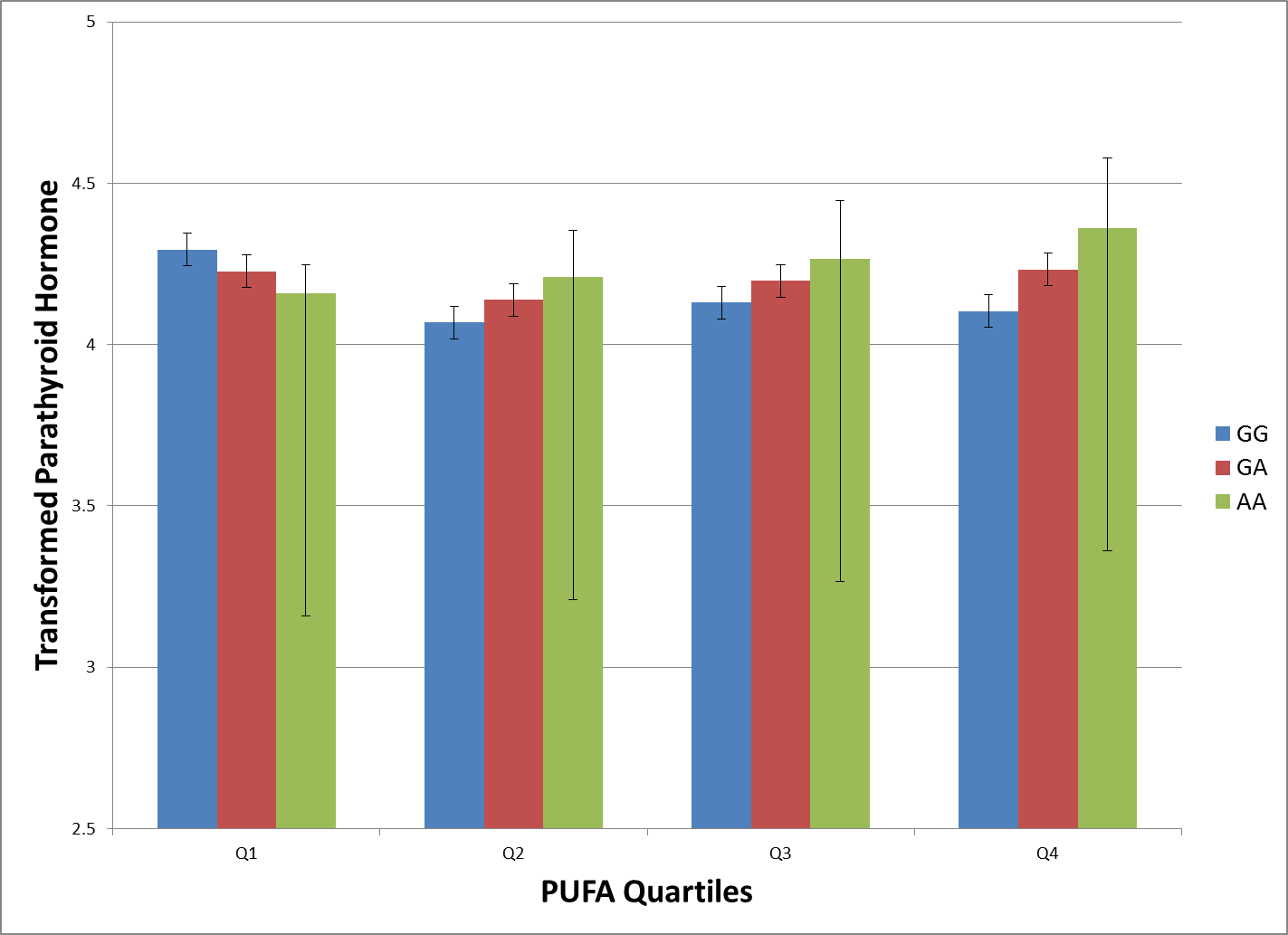

Supplement: Additional file 2: Figure S1. — Distribution of predicted mean values (±standard deviation) of transformed 25(OH)D levels for rs10205487 genotypes within n-3 PUFA quartiles (n = 924). The X-axis shows the n-3 PUFA quartiles, and the Y-axis shows Box-Cox transformed 25(OH)D levels, with the colors of the bars corresponding to the genotype. (DOCX 68.9 kb) [file 12263_2016_538_MOESM2_ESM.docx]
