# Supplementary material for: Linkage and association analysis of circulating vitamin D and parathyroid hormone identifies novel loci in Alaska Native Yup’ik people
Source: Genes Nutr. 2016 Aug 2;11:23. doi: 10.1186/s12263-016-0538-y (PMC4971612; doi:10.1186/s12263-016-0538-y)
Supplement: Supplementary file 3 [file 12263_2016_538_MOESM3_ESM.docx]

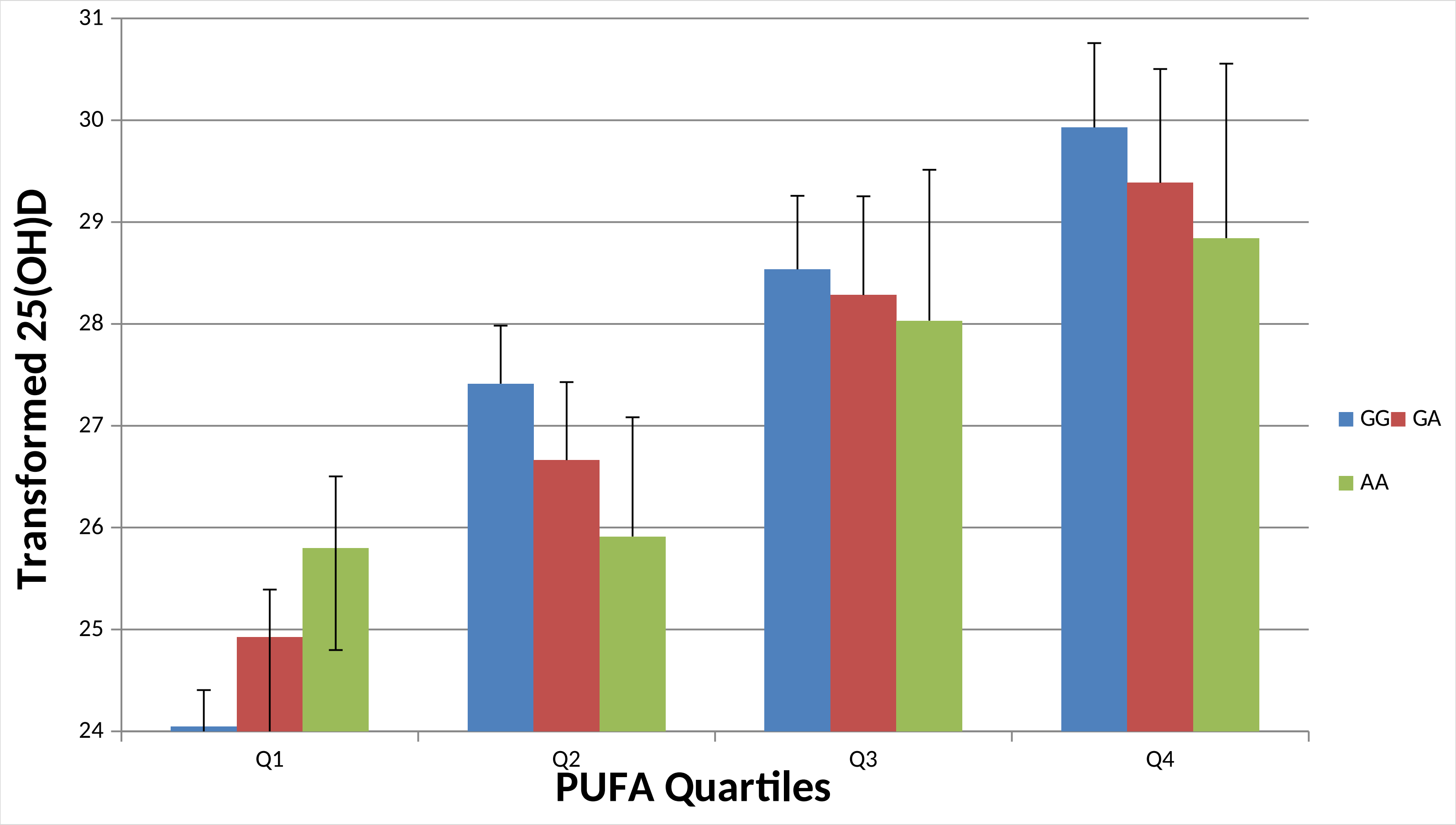

31
30
29
28
27
26
25
24
Transformed 25(OH)D
Q1
Q2
Q3
Q4
PUFA Quartiles
GG
GA
AA
